# Supplementary material for: Heat adaptation of phage T7 under an extended genetic code
Source: Virus Evol. 2021 Dec 1;7(2):veab100. doi: 10.1093/ve/veab100 (PMC8923235; doi:10.1093/ve/veab100)
Supplement: veab100_Supp [file veab100_supp.zip › Sequencing_Analysis.zip!/Sequencing_Analysis/A3/evidence/JC_SIDE_1_66.html]

BRESEQ :: Evidence


| Predicted mutation | | | | | | |
| --- | --- | --- | --- | --- | --- | --- |
| evidence | seq id | position | mutation | annotation | gene | description |
| MC JC | NC\_001604.1 | 13,278 | Δ1,019 bp | intergenic (–/–) | – / – | –/– |

| Missing coverage evidence... | | | | | | | | | | |
| --- | --- | --- | --- | --- | --- | --- | --- | --- | --- | --- |
|  |  |  | seq id | start | end | size | ←reads | reads→ | gene | description |
| \* | \* | ÷ | NC\_001604.1 | 13278 | 14296 | 1019 | 1612 [523] | [14] 1100 | *–/–* | –/– |

| New junction evidence | | | | | | | | | | | |
| --- | --- | --- | --- | --- | --- | --- | --- | --- | --- | --- | --- |
|  | | seq id | position | reads (cov) | reads (cov) | score | skew | freq | annotation | gene | product |
| \* | ? | NC\_001604.1 | = 13277 | 522 (0.300) | 1073 (0.630) | 291/586 | 0.3 | 80.4% | intergenic (–/–) | *–/–* | –/– |
|  | ? | NC\_001604.1 | 14297 = | 14 (0.010) |  |  |  |  | intergenic (–/–) | *–/–* | –/– |

Only 100 of 1612 total aligned reads displayed.  

`AGGTAAAGCACATGAGGAAGGTCGCCCCGTTTCTATTACTGACCTACGTGGTTCTGGCGCACTACGCCAACTATCTGATACTATTATTGCCCTTGAGCGTAATCAGCAAGGCGATATGCCTAACCTTGTCCTCGTTCGTATTCTCAAGTGCCGCTTTACTGGTGATACTGGTATCGCTGGCTACATGGAATACAACAAGGAAACCGGATGGCTTGAACCATCAAGTTACTCAGGGGAAGAAGAGTCACACTCAGAGTCAACAGACTGGTCCAACGACACTGACTTCTGACAGGATTCTTGATGACTTTCCAGACGACTACGAGAAGTTTCGCTGGAGAGTCCCATTCTAATACGACTCACTAAAGGAGACACACCATGTTCAAACTGATTAAGAAGTTAGGCCAACTGCTGGTTCGTATGTACAACGTGGAAGCCAAGCGACTGAACGATGAGGCTCGTAAAGAGGCCACACAGTCACGCGCTCTGGCGATTCGCTCCAACGAACTGGCTGACAGTGCATCCACTAAAGTTACCGAGGCTGCCCGTGTGGCAAACCAAGCTCAACAGCTTTCCAAATTCTTTGAGTAATCAAACAGGAG  >  NC_001604.1/12977‑13575`  

                                                                                                                                                                                                                                                                                                            `|`                                                                                                                                                                                                                                                                                                            
  `gTAAAGCACATGAGGAAGGTCGCCCAGTTTCTATTACTGACCTACGTGGTTCTGGCGCACTACGCCAACTATCTGATACTATTATTGCCCTTGAGCGTAATCAGCAAGGCGATATGCCTAACCTTGTCATCGTTCGTATTCTCAAGTGACGCTTTACTGGTGATACTGGTATCGCTGGCTACATGGAATACAACAAGGAAACCGGATGGCTTGAACCATCAAGATACTCAGGGGAAGAAGAGTCACACTAAGAGTAAACAGACTGGTCCAACGACACGGACTTCTGACAGGATTCATGAaa                                                                                                                                                                                                                                                                                                          >  1:17919‑M1/1‑299 (MQ=255)`  
      `aGCACATGAGGAAGGTCGCCCCGTTTCTATTACTGACCTACGTGGTTCTGGCGCACTACGCCAACTATCTGATACTATTATTGCCCTTGAGCGTAATCAGCAAGGCGATATGCCTAACCTTGTCCTCGTTCGTATTCTCAAGTGCCGCTTTACTGGTGATACTGGTATCGCTGGCTACATGGAATACAACAAGGAAACCGGATGGCTTGAACCATCAAGTTACTCAGGGGAAGAAGAGTCACACTCAGAGTCAACAGACTGGTCCAACGACACTGACTTCTGACAGGTTTCTTGAcagttg                                                                                                                                                                                                                                                                                                      <  1:62332‑M1/301‑7 (MQ=255)`  
       `gCACATGAGGAAGGTCGCCCCGTTTCTATTACGGACCTACGTGGTTCTGGCGCACTACGCCAACTATCTGATACTATTATTACCCTTGAGCGTAATCAGCAAGGCGATATGCCTAACCTTGTCCTCGTTCGTATTCTCAAGTGCCGCTTTACTGGTGATACTGGTATCGCTGGCTACATGGAATACAACAAGGAAACCGGATGGCTTGAACCATCAAGTTACTCAGGGGAATAAGAGTCACACTCAGAGTCAACAGACTGGTCCAACGACACTGACTTCTGACAGGATTCTTGCTGACttt                                                                                                                                                                                                                                                                                                     >  1:23001/1‑301 (MQ=255)`  
         `gagatgtgtataagagacagTTTCTAGTACTGACCTACGTGGTTCTGGCGCACTACGCCAACTATCTGATACTATTATTGCCCTTGAGCGTAATCAGCAAGGCGATATGCCTAACCTTGTCCTCGTTCGTATTCTCAAGTGCCGCTTTACTGGTGATACTGGTATCGCTGGCTACATGGAATACAACAAGGAAACCGGATGGCTTGAACCATCAAGTTACTCAGGGGAAGAAGAGTCACACTCAGAGTCAACAGACTGGTCCAACGACACTGACTTCTGACAGGATTCTTGATGACTTTcc                                                                                                                                                                                                                                                                                                   <  1:233688/282‑1 (MQ=255)`  
         `agaTGAGGAAGGTCGCCCCGTTTCTATTACGGACCTACGTGGTTCTGGCGCACTACGCCAACCATCTGATACTATTATTGCCCTTGAGCGTAATCAGCAAGGCGATATGCCTAACCTTGTCCTCGTTCTTATTCTCAAGTGCCGATTTACTGGTGATACTGGTATCGCTGGCTACATGGAATACAACAAGGAAACCGGATGGCTTGAACCATCAAGTTACTCAGGGGAAGAAGAGTCACACTCAGAGTCAACAGACTGGTCCAACGACACTGACTTCTGACAGGATTCTTGATGACTGTcc                                                                                                                                                                                                                                                                                                   <  1:206987/299‑1 (MQ=255)`  
                `gAAGGTCGCCCCGTTTCTATTACTGACCTACGTGGTTCTGGCGCACTACGCCAACTATCTGATACTATTATTGCCCTTGAGCGTAATCAGCAAGGCGATATGCCTAACCTTGTCCTCGTTCGTATTCTCAAGTGCCGCTTTACTGGTGATACTGGTATCGCTGGCTACATGGAATACAACAAGGAAACCGGATGGCTTGAACCATCAAGTTACTCAGGGGAAGAAGAGTCACACTCAGAGTCAACAGACTGGTCCAACGACACTGACTTCTGACAGGATTCTTGATGACTTTCCAgacgac                                                                                                                                                                                                                                                                                            <  1:16629/301‑1 (MQ=255)`  
                             `tttCTATTACTGACCTACGTGGTTCTGGCGCACTACGCCAACTATCTGATACTATTATTGCCCTTGAGCGTAATCAGCAAGGCGATATGCCTAACCTTGTCCTCGTTCGTATTCTCAAGTGCCGCTTTACTGGTGATACTGGTATCGCTGGCTACATGGAATACAACAAGGAAACCGGATGGCTTGAACCATCAAGTTACTCAGGGGAAGAAGAGTCACACTCAGAGTCAACAGACTGGTCCAACGACACTGACTTCTGACAGGATTCTTGATGACTTTCCAGACGACTACGAGAAGTTTc                                                                                                                                                                                                                                                                               >  1:131111/1‑301 (MQ=255)`  
                              `ttCTATTACTGACCTACGTGGTTCTGGCGCACTACGCCAACTATCTGATACTATTATTGCCCTTGAGCGTAATCAGCAAGGCGATATGCCTAACCTTGTCCTCGTTCGTATTCTCAAGTGCAGCTTTACTGGTGATACTGGTATCGCTGGCTACATGGAATACAACAAGGAAACCGGATGGCTTGAACCATCAAGTTACTCAGGGGAAGAAGAGTCACACTCAGAGTCAACAGACTGGTCCAACGACACTGACTTCTGACAGGATTCTTGAcagttgtttcatatgaagagattgttaagt                                                                                                                                                                                                                                                                              >  1:122035‑M1/1‑271 (MQ=255)`  
                              `ttCTATTACTGACCTACGTGGTTCTGGCGCACTACGCCAACTATCTGATACTATTATTGCCATTGAGCGTAATCAGCAAGGCGATATGCCTAACCTTGTCCTCGTTCGTATTCTCAAGTGCCGCTTTACTGGTGATACTGGTATCGCTGGCTACATGGAATACAACAAGGAAACCGGATGGCTTGAACCATCAAGTTACTCAGGGGAAGAAGAGTCACACTCAGAGTCAACAGACTGGTCCAACGACACTGACTTCTGACAGGATTCTTGATGACTTTCCAGACGACTACGAGAAGTTTcc                                                                                                                                                                                                                                                                              >  1:320220/1‑300 (MQ=255)`  
                                `gtataagagacagacgtggttctggcgCACTACGCCAACTATCTGATACTATTATTGCCCTTGAGCGTAATCAGCAAGGCGATATGCCTAACCTTGTCCTCGTTCGTATTCTCAAGTGCCGCTTTACTGGTGATACTGGTATCGCTGGCTACATGGAATACAACAAGGAAACCGGATGGCTTGAACCATCAAGTTACTCAGGGGAAGAAGAGTCACACTCAGAGTCAACAGACTGGTCCAACGACACTGACTTCTGACAGGATTCTTGAcagttgtttcatatgaagagattgttaagtca                                                                                                                                                                                                                                                                            <  1:7157‑M1/288‑33 (MQ=255)`  
                                   `ttACTGACCTACGTGGTTCTGGCGCACTACGCCAACTATCTGATACTATTATTGCCCTTGAGCGTAATCAGCAAGGCGATATGCCTAACCTTGTCCTCGTTCGTATTCTCAAGTGCCGCTTTACTGGTGATACTGGTATCGCTGGCTACATGGAATACAACAAGGAAACCGGATGGCTTGAACCATCAAGTTACTCAGGGGAAGAAGAGTCACACTCAGAGTCAACAGACTGGTCCAACGACACTTACTTCTGACAGGATTCTTGAtagttgtttcatatgaagagattgttaagtcacga                                                                                                                                                                                                                                                                         >  1:30215‑M1/1‑266 (MQ=255)`  
                                   `ttACTGACCTACGTGGTTCTGGCGCACTACGCCAACTATCTGATACTATTATTGCCCTTGAGCGTAATCAGCAAGGCGATATGCCTAACCTTGTCCTCGTTCGTATTCTCAAGTGCCGCTTTACTGGTGATACTGGTATCGCTGGCTACATGGAATACAACAAGGAAACCGGATGCCTTGAACCATCAAGTTACTCAGGGGAAGAAGAGTCACACTCAGAGTCAACAGACTGGTCCAACGACACTGCCTTCTGACCGGATTCTTGAcagttgtttcatatgaatagcttgttaagtcacga                                                                                                                                                                                                                                                                         >  1:148598‑M1/1‑266 (MQ=255)`  
                                        `gACCTACGTGGTTCTGGCGCACTACGCCAACTATCTGATACTATTATTGCCCTTGAGCGTAATCAGCAAGGCGATATGCCTAACCTTGTCCTCGTTCGTATTCTCAAGTGCCGCTTTACTGGTGATACTGGTCTCGCTGGCTACATGGAATACAACAAGGAAACCGGATGGCTTGAACCATCAAGTTACTCATGGGAAGAAGAGTCACACTCAGAGTCAACAGACTGGTCCAACGACACTGACTTCTGACAGGATTCTTGATGACTTTCCATACGACTACGAGAAGTTTCGCTGTAGTGTc                                                                                                                                                                                                                                                                    >  1:44497/1‑301 (MQ=255)`  
                                                          `gcACTACGCCAACTATCTGATACTATTATTGCCCTTGAGCGTAATCAGCAAGGCGATATGCCTAACCTTGTCCTCGTTCGTATTCTCAAGTGCCGCTTTACTGGTGATACTGGTATCGCTGGCTACATGGAATACAACAAGGAAACCGGATGGCTTGAACCATCAAGTTACTCAGGGGAAGAAGAGTCACACTCAGAGTCAACAGACTGGTCCAACGACACTGACTTCTGACAGGATTCTTGAcagttgtttcatatgaagagattgttaagtcacgataatcaataggagaaatcaatat                                                                                                                                                                                                                                                  >  1:91755‑M1/1‑243 (MQ=255)`  
                                                             `cTACGCCAACTATCTGATACTATTATTGCCCTTGAGCGTAATCAGCAAGGCGATATGCCTAACCTTGTCCTCGTTCGTATTCTCAAGTGCCGCTTTACTGGTGATACTGGTATCGCTGGCTACATGGAATACAACAAGGAAACCGGATGGCTTGAACCATCAAGTTACTCAGGGGAAGAAGAGTCACACTCAGAGTCAACAGACTGGTCCAACGACACTGACTTCTGACAGGATTCTTGATGACTTTCCAGACGACTACGAGAAGTTTCGCTGGAGAGTCCCATTCTAATACGACTCACTa                                                                                                                                                                                                                                               >  1:88161/1‑301 (MQ=255)`  
                                                             `cTACGCCAACTATCTGATACTATTATTGCCATTGAGCGTAATCAGCAAGGCGATATGCCTAACCTTGTCCTCGTTCGTATTCTCAAGTGCCGCTTTACTGGTGATACTGGTATCGCTGGCTACATGGAATACAACAAGGAAACCGGATGGCTTGAACCATCAAGTTACTCAGGGGAAGAAGAGTCACACTCAGAGTCAACAGACTGGTCCAACGACACTGACTTCTGACAGGATTCTTGAcagttgtttcatatgaagagattgttaagtcacgataatcaataggagaaatcaatatgat                                                                                                                                                                                                                                               <  1:163126‑M1/301‑62 (MQ=255)`  
                                                                   `cagcTATCTGATACTATTATTGCCCTTGAGCGTAATCAGCAAGGCGATATGCCTAACCTTGGCCTCGTTCGTATTCTCAAGTGCCGCTTTACTGGTGATACTGGTATCGCTGGCTACATGGAATACAACAAGGAAACCGGATGGCTTGAACCATCAAGTTACTCAGTGGAAGAAGAGTCACACTCAGAGTCAACAGACTGGTCCAACGACACTGACTCCTGACAGGATTCTTGATGACTTTCCAGACGACTACGAGAAGTTTCGCTGGAGAGTCCCATTCTAATACGACTCACTAAAGgag                                                                                                                                                                                                                                         <  1:24112/298‑1 (MQ=255)`  
                                                                       `tATCTGATACTATTATTGCCCTTGAGCGTAATCAGCAAGGCGATATGCCTAACCTTATCCTCGTTCGTATTCTCAAGTGCCGCTTTACTGGTGATACTGGTATCGCTGGCTACATGGAATACAACAAGGAAACCGGATGGCTTGAACCATCAAGTTACTCAGGGGAAGAAGAGTCACACTCAGAGTCAACAGACTGGTCCAACGACACTGACTTCTGACAGGATTCTTGAcagttgtttcatatgaagagattgttaagtcacgataatcaataggagaaatcaatatgatcgtttctgac                                                                                                                                                                                                                                     <  1:4735‑M1/301‑72 (MQ=255)`  
                                                                        `aTCTGATACTATTATTGCCCTTGAGCGTAATCAGCAAGGCGATATGCCTAACCTTGTCCTCGTTCGTATTCTCAAGTGCCGCTTTACTGGTGATACTGGTATCTCTGGCTACATGGAATACAACAAGGAAACCGGATGGCTTGAACCATCAAGTTACTCAGGGGAAGAATAGTCACACTCAGAGTCAACAGACTGGTCCAACGACACTGACTTCTGACAGGATTCTTGATGACTTTCCAGACGACTACGAGAAGTTTCGCTGGAGAGTCCCATTCTAATACGACtgtctcttatacacctt                                                                                                                                                                                                                                    >  1:155987/1‑285 (MQ=255)`  
                                                                        `aTCTGATACTATTATTGCCCTTGAGCGTAATCAGCAAGGCGATATGCCTAACCTTGTCCTCGTTCGTATTCTCAAGTGCCGCTTTACTGGTGATACTGGTATCGCTGGCTACATGGAATACAACAAGGAAACCGGATGGCTTGAACCATCAAGTTACTCCGGGGAAGAAGAGTAACACTCAGAGTCAACAGACTGGTCCAACGACACTGACTTCTGACAGGATTCTTGATGACTTTCCAGACGACTACGAGAAGTTTCGCTGGAGAGTCCCATTCTAATACGAcctgtctcttatacacat                                                                                                                                                                                                                                    >  1:124829/1‑284 (MQ=255)`  
                                                                        `aTCTGATACTATTATTGCCCTTGAGCGTAATCAGCAAGGCGATATGCCTAACCTTGTCCTCGTTCGTATTCTCAAGTGCCGCTTTACTGGTGATACTGGTATCGCTGGCTACATGGAATACAACAAGGAAACCGGATGGCTTGAACCATCAAGTTACTCAGGGGAAGAAGAGTCACACTCAGAGTCAACAGACTGGTCCAACGACACTGACTTCTGACAGGATTCTTGAcagttgtttcatatgaagagattgttaagtcacgataatcaataggagaaatcaatattatcgtttctgaca                                                                                                                                                                                                                                    >  1:209901‑M1/1‑229 (MQ=255)`  
                                                                        `aTCTGATACTATTATTGCCCTTGAGCGTAATCAGCAAGGCGATATGCCTAACCTTGTCCTCGTTCGTATTCTCAAGTGCCGCTTTACTGGTGATACTGGTATCGCTGGCTACATGGAATACAACAAGGAAACCGGATGGCTTGAACCATCAAGTTACTCAGGGGAAGAAGAGTCACACTCAGAGTAAACAGACTGGTCCAACGACACTGACTTCTGACAGGATTCTTGAaagttgtttcatatgaagagattgttaagtcacgataatcaataggagctgtctcttatacacatctacgag                                                                                                                                                                                                                                    >  1:31475‑M1/1‑229 (MQ=255)`  
                                                                        `aTCTGATACTATTATTG‑CCTTGAGCGTAATCAGCAAGGCGATATGCCTAACCTTGTCCTCGTTCGTATTCTCAAGTGCCGCTTTACTGGTGATACTGGTATCGCTGGCTACATGGAATACAACAAGGAAACCGGATGGCTTGAACCATCAAGTTACTCAGGGGAAGAAGAGTCACACTCAGAGTCAACAGACTGGTCCAACGACACTGACTTCTGACAGGATTCTTGATGACTTTCCAGACGACTACTAGAAGTTTCGCTGGAGAGTCCCATTcctgtctcttatacacatctccgagccc                                                                                                                                                                                                                                   >  1:174273/1‑274 (MQ=255)`  
                                                                         `aagagacagattattGCCCTTGAGCGTAATCAGCAAGGCGATATGCCTAACCTTGTCCTCGTTCGTATTCTCAAGTGCCGCTTTACTGGTGATACTGGTATCGCTGGCTACATGGAATACAACAAGGAAACCGGATGGCTTGAACCATCAAGTTACTCAGGGGAAGAAGAGTCACACTCAGAGTCAACAGACTGGTCCAACGACACTGACTTCTGACAGGATTCTTGATGACTTTCCAGACGACTACGAGAAGTTTCGCTGGAGAGTCCCATTCTAATACGACTCACTAAAGGAGacacac                                                                                                                                                                                                                                   <  1:133342/292‑1 (MQ=255)`  
                                                                          `cTGATACTATTATTGCCCTTGAGCGTAATCAGCAAGGCGATATGCCTAACCTTGTCCTCGTTCGTATTCTCAAGTGCCGCTTTACTGGTGATACTGGTATCGCTGGCTACATGGAATACAACAAGGAAACCGGATGGCTTGAACCATCAAGTTACTCAGGGGAAGAAGAGTCACACTCAGAGTCAACAGACTGGTCCAACGACACTGACTTCTGACAGGTTTCTTGAcagttgtttcatatgaagagattgttaagtcacgataatcaataggagaaatcaatctgtctcttatacacatc                                                                                                                                                                                                                                  >  1:294891‑M1/1‑227 (MQ=255)`  
                                                                          `cTGATACTATTATTGCCCTTGAGCGTAATCAGCAAGGCGATATGCCTAACCTTGTCCTCGTTCGTATTCTCAAGTGACGCTTTACTGGTGCTACTGGTATCGCTGGCTACATGGAATACAACAAGGAAACCGGATGGCTTGAACCATCAAGTTACTCAGGGGAAGAAGAGTCACACTCAGAGTCAACAGACTGGTCCAACGACACTGACTTCTGACAGGATTCTTGATGACTTTCCAGACGACTACGAGAAGTTTCGCTGGAGAGTCCCATTCTCATACGACTCACTAAAGGCGacctgtc                                                                                                                                                                                                                                  >  1:74131/1‑296 (MQ=255)`  
                                                                              `tACTATTATTGCCCTTGAGCGTAATCAGCAAGGCGATATGCCTAACCTTGTCCTCGTTCGTATTCTCAAGTGCCGCTTTACTGGTGATACTGGTATCGCTGGCTACATGGAATACAACAAGGAAACCGGATGGCTTGAACCATCAAGTTACTCAGGGGAAGAAGAGTCACACTCAGAGTCAACAGACTGGTCCAACGACACTGACTTCTGACAGGATTCTTGAcagttgtttcatatgaagagattgttaagtcacgataatcaataggagaaatcaatatgatcgtttctgacatcgaag                                                                                                                                                                                                                              >  1:58077‑M1/1‑223 (MQ=255)`  
                                                                               `aCTATTATTGCCCTTGAGCGTAATCAGCAAGGCGATATGCCTAACCTTGTCCTCGTTCGTATTCTCAAGTGCCGCTTTACTGGTGATACTGGTATCGCTGGCTACATGGAATACAACAAGGAAACCGGATGGCTTGAACCATCAAGTTACTCAGGGGAAGAAGAGTCACACTCAGAGTCAACAGACTGGTCCAACGACACTGACTTCTGACAGGATTCTTGAcagttgtttcatatgaagagattgttaagtcacgataatcaataggagaaatcaatatgatcgtttctgacatcgaagc                                                                                                                                                                                                                             <  1:201152‑M1/301‑80 (MQ=255)`  
                                                                                `cTATTATTGCCCTTGAGCGTAATCAGCAAGGCGATATGCCTAACCTTGTCCTCGTTCGTATTCTCAAGTGCCGCTTTACTGGTGATACTGGTATCGCTGGCTACATGGAATACAACAAGGAAACCGGATGGCTTGAACCATCAAGTTACTCAGGGGAAGAAGAGTCACACTCAGAGTCAACAGACTGGTCCAACGACACTGACTTCTGACAGGCTTCTTGAcagttgtttcatatgaagagattgttaagtcacgataatcaataggagaaatcaatctgatcgtttctgacatcgaatct                                                                                                                                                                                                                            >  1:322700‑M1/1‑221 (MQ=255)`  
                                                                                `cTATTATTGCCCTTGAGCGTAATCAGCAAGGCGATATGCCTAACCTTGTCCTCGTTCGTATTCTCAAGTGCCGCTTTACTGGTGATACTGGTATCGCTGGCTACATGGAATACAACAAGGAAACCGGATGGCTTGAACCATCAAGTTACTCAGGGGAAGAAGAGTCACACTCAGAGTCAACAGACTGGTCCAACGACACTGACTTCTGACAGGATTCTTGATGACTTTCCAGACGACTACGAGAAGTTTCGCTGGAGAGTCCCATTCTAATACGACTCACTAAAGGAGACACACCATGATc                                                                                                                                                                                                                            >  1:264553/1‑301 (MQ=255)`  
                                                                                 `tattatTGCCCTTGAGCGGAATCAGCAAGGCGATATGCCTAACCTTGTCCTCGTTCGTATTCTCAAGTGCCGCTTTACTGGTGATACTGGTATCGCTGGCTACATGGAATACAACAAGGAAACCGGATGGCTTGAACCATCAAGTTACTCAGGGGAAGAAGAGTCACACTCAGAGTCAACAGACTGGTCCAACGACACTGACTTCTGACAGGATTCTTGATGACTTTCCAGACGACTACGAGAAGTTTCGCTGGAGAGTCCCATTCTAATACGACTCACTAAAGGAGACACACCATGATcg                                                                                                                                                                                                                           <  1:10875/301‑2 (MQ=255)`  
                                                                                  `attattGCCCTTGAGCGTAATCAGCAAGGCGATATGCCTAACCTTGTCCTCGTTCGTATTCCCAAGTGCCGCTTTACTGGTGATACTGGTATCGCTGGCTACATGGAATACAACAAGGAAACCGGATGGCTTGAACCATCAAGTTACTCAGGGGAAGAAGAGTCACACTCAGAGTCAACAGACTGGTCCAACGACACTGACTTCTGACAGGATTCTTGAcagttgtttcatatgaagagattgttaagtcacgataataaaacaggagaaaccattatgatcgtttctgacatcgaagcta                                                                                                                                                                                                                          >  1:51696‑M1/1‑219 (MQ=255)`  
                                                                                    `tattGCCCTTGAGCGTAATCAGCAAGGAGATATGCCTAACCTTGTCCTCGTTCGTATTCTCAAGTGCCGCTTTACTGGTGATACTGGTATCGCTGGCTACATGGAATACAACAAGGAAACCGGATGGCTTGAACCATCAAGTTACTCAGGGGAAGAAGAGTCATACTCAGAGTCAACAGACTGGTCCAACGACACTGACTTCTGACAGGATTCTTGATGACTTTCCAGACGACTACGAGAAGTTTCGCTGGAGAGTCCCATTCTAATACGACTCACTAAAGGAGACACACCATGTTCAAAc                                                                                                                                                                                                                        <  1:139881/301‑1 (MQ=255)`  
                                                                                              `gAGCGTAATCAGCAAGGCGATATGCCTAACCTTTTCCTCGTTCGTATTCTCAAGTGCCGCTTTACTGGTGATACTGGTATCGCTGGCTACCTGGAATACAACCAGGAAACCGGATGGCTTGAACCATCAAGTTACTCAGGGGAAGACGAGTCACACTCAGAGTCAACAGACTGGTCCAACGACACTGACTTCTGACAGGATTCTTTATGACTTTCCAGACGACTACGAGAAGTTTCGCTGGAGAGTCCCCTTCTAATACGACTCACTAAAGGAGACACACCATTTTCAAACTGATTacaca                                                                                                                                                                                                              >  1:335724/1‑297 (MQ=255)`  
                                                                                              `gAGCGTAATCAGCAAGGCGATATGCCTAACCTTGTCCTCGTTCGTATTCTCAAGTGCCGCTTTACTGGTGATACTGGTATCGCTGGCTACATGGAATACAACAAGGAAACCGGATGGCTTGATCCATCAAGTTACTCAGGGGAAGAAGAGTCACACTCAGAGTCAACAGACTGGTCCAACGACACTGACTTCTGACAGGATTCTTGAcagttgtttcatatgaagagattgttaagtcacgataatcaataagagaaatcaatatgatcgtttctgacatcgaagctaacgccctcttaga                                                                                                                                                                                                              >  1:100119‑M1/1‑207 (MQ=255)`  
                                                                                               `tgtataagagacagtgcgatatgcctaacctTGACCTCGTTCGTATTCTCAAGTGCCACTTTACTGGTGATACTGGTATCGCTGGCTACATGGAATACAACAATGAAACCGTATGGCTTGAACCATCAAGTTACTCAGGGGAAGAAGAGTCACACTCAGAGTCAACAGACTGGTCCAACGACACTGACTTCTGACAGGATTCTTGAcagttgtttcatatgaagagattgttaagtcacgataatcaataggagaaatcaatatgatcgtttctgacatcgaagctaacgccctcttagag                                                                                                                                                                                                             <  1:81511‑M1/286‑96 (MQ=255)`  
                                                                                               `agagacagcagcaaggcGATATGCCTAACCTTGTCCTCGTTCGTATTCTCAAGTGCCGCTTTACTGGTGATACTGGTATCGCTGGCTACATGGAATACAACAAGGAAACCGGATGGCTTGAACCATCAAGTTACTCAGGGGAAGAAGAGTCACACTCAGAGTCAACAGACTGGTCCAACGACACTGACTTCTGACAGGATTCTTGAcagttgtttcatatgaagagattgttaagtcacgataatcaataggagaaatcaatatgatcgtttctgacatcgaagctaacgccctcttagag                                                                                                                                                                                                             <  1:55726‑M1/293‑96 (MQ=255)`  
                                                                                               `aGCGTAATCAGCAAGGCGATATGCCTAACCTTGTCCTCGTTCGTATTCTCAAGTGCCGCTTTACTGGTGATACTGGTATCGCTTGCTACATGGAATACAACAAGGAAACCGGATGGCTTTAACCATCAAGTTACTCAGGGGAAGAAGAGTCACACTCAGAGTCAACAGACTGGTCCAACGACACTGACTTCTGACAGGATTCTTGAcagttgtttcatatgaagagattgttaagtcacgataatcaataggagaaatcaatatgatcgtttctgacatcgaagctaacgccctcttagag                                                                                                                                                                                                             <  1:105511‑M1/301‑96 (MQ=255)`  
                                                                                               `aGCGTAAACAGCAAAGCGATATGCCTAACCTTGTCCTCGTTCGTATTATCAAGTGCCGCGTTACTGGAGATACTGGTATCGCTGGCTACATGGAATACAACAAGGAAACCGGATGGCTTGAACCATCAAGTTACGCTGGGGAAGAAGAGTCACACTCAGAGTCAACAGACTGGTCCAACGACACTGACTTCTGACAGGATTCTTGAcagttgtttcatatgaagagattgttaagtcacgataatcaataggagaaatcaatatgatcgtttctatcatcgaagctaacgccctcttagag                                                                                                                                                                                                             <  1:42046‑M1/301‑96 (MQ=255)`  
                                                                                                 `ggtAATCAGCAAGGCGATATGCATAACCTTGTGCTCGTTCGTATTCTCAAGTGCCGCTTTACTGGTGATACTGGTATCGCTGGCTACATGGAATACAACAAGGAAACCGGATGGCTTGAACCATCAAGTTACTCAGGGGAAGAAGAGTCACACTCAGAGTCAACAGACTGGTCCAACGACACTGACTTCTGACAGGATTCTTGAcagttgtttcatatgaagagattgttaagtcacgataatcaataggagaaatcaatatgatcgtttctgacatcgaagctaacgccctcttagagag                                                                                                                                                                                                           <  1:288094‑M1/300‑98 (MQ=255)`  
                                                                                                  `gTAATCAGCAATGCGATATGCCTAACCTTGTCCTCGTTCGTATTCTCAAGTGCCGCTTTACTGGTGATACTGGTATCGCTGGCTACATGGAATACAACAAGGAAACCGGATGGCTTGAACCATCAAGTTACTCAGGGGAAGAAGAGTCACACTCAGAGTCAACAGACTGGTCCAACGACACTGACTTCTGACAGGATTCTTGATGACTTTCCAGACGACTACGAGAAGTTTCGCTGGAGAGTCCCATTCTAATACGACTCACTAAAGGAGACACACCATGATcgtttctgacatcgaagct                                                                                                                                                                                                          >  1:177206/1‑283 (MQ=255)`  
                                                                                                  `agatgtgtataagagacaggcctaaccttgtcctcgttcGTATTCTCAAGTGCCGCTTTACTGGTGATACTGGTATCGCTGGCTACATGGAATACAACAAGGAAACCGGATGGCTTGAACCATCAAGTTACTCAGGGGAAGAAGAGTCACACTCAGAGTCAACAGACTGGTCCAACGACACTGACTTCTGACAGGATTCTTGAcagttgtttcatatgaagagattgttaagtcacgataatcaataggagaaatcaatatgatcgtttcttacatcgaagctaacgccctcttagagagc                                                                                                                                                                                                          <  1:185970‑M1/282‑99 (MQ=255)`  
                                                                                                    `aaTCAGCAAGGCGATATGCCTAACCTTGTCCTCGTTCGTATTCTCAAGTGCCGCTTTACTGGTGATACTGGTATCGCTGGCTACATGGAATACAACAAGGAAACCGGATGGCTTGAACCATCAAGTTACTCAGGGGAAGAAGAGTCACACTCAGAGTCAACAGACTGGTCCAACGACACTGACTTCTGACAGGATTCTTGAcagttgtttcatatgaagagattgttaagtcacgataatcaataggagaaatcaatatgatcgtttctgacatcgaagctaacgacctcttagagagcgt                                                                                                                                                                                                        >  1:220996‑M1/1‑201 (MQ=255)`  
                                                                                                         `gCAAGGCGATATGCCTAACCTTGTCCTCGTTCGTATTCTCAAGTGCCGCTTTACTGGTGATACTGGTATCGCTGGCTACATGGAATACAACAAGGAAACCGGATGGCTTGAACCATCAAGTTACTCAGGGGAAGAAGAGTCACACTCAGAGTCAACAGACTGGTCCAACGACACTGACTTCTGACAGGATTCTTGAcagttgtttcatatgaagagattgttaagtcacgataatcaataggagaaatcaatatgatcgtttctgacatcgaagctaacgccctcttagagagcgtaacta                                                                                                                                                                                                   >  1:65626‑M1/1‑196 (MQ=255)`  
                                                                                                               `cGATATGCCTAACCTTGTCCTCGTTCATATTCTCAAGTGCCGCTTTACTGGTGATACTGGTATCGCTGGCTACATGGAATACAACAAGGAAACCGGATGGCTTGAACCATCAAGTTACTCAGGGGAAGAAGAGTCACACTCAGAGTCAACAGACTGGTCCAACGACACTGACTTCTGACAGGATTCTTGATGACTTTCCAGACGACTACGAGAAGTTTCGCTGGAGAGTCCCATTCTAATACGACTCACTAAAGGAGACACACCATGTTCAAACTGATTAAGAAGTTAGGcctgtctctta                                                                                                                                                                                             >  1:48806/1‑292 (MQ=255)`  
                                                                                                                 `atatGCCTAACCTTGTCCTCGTTCGTATTCTCAAGTGCCGCTTTACTGGTGATACTGGTATCGCTTGCTACATGGAATACAACAAGGAAACCGGATGGCTTGAACCATCAAGTTACTCAGTGGAAGAAGAGTCACACTCAGAGTCAACAGACTGGTCCAACGATACTGACTTCTGACAGGATTCTTGAcagttgtttcatatgaagagattgttaagtcacgataatcaataggagaaatcaatatgatcgtttctgacatcgaagctaacgccctcttagagagcgtcactaagttccac                                                                                                                                                                                           <  1:137619‑M1/301‑114 (MQ=255)`  
                                                                                                                   `atGCCTAACCTTGTCCTCGTTCGTATTCTCAAGTGCCGCTATACTGGTGATACTGGTATCGCTGGCTACATGGAATACAACAAGGAAACCGGATGGCTTGAACCATCAAGTTACTCAGGGGAAGAAGAGTCACACTCAGAGTCAACAGACTGGTCCAACGACACTGACTTCTGACAGGATTCTTGAcagttgtttcatatgaagagattgttaagtcacgataatcaataggagaaatcaatatgatcgtttctgacatcgaagctaacgcactcttagagagcgtcactaagttccactg                                                                                                                                                                                         >  1:298013‑M1/1‑186 (MQ=255)`  
                                                                                                                    `tGCCTAACCTTGTCCTCGTTCGTATTCTCAAGTGCCGCTTTACTGGTGATACTGGTATCGCTGGCTATATGGAATACAACAAGGAAACCGGATGGCTTGAACCATCAAGTTACTCAGGGGAAGAAGAGTCACACTCAGAGTCAACAGACTGGTCCAACGACACTGACTTCTGACAGGATTCTTGAcagttgtttcatatgaagagattgttaagtcacgataatcaataggagaaatcaatatgatcgtttctgacatcgaagctaacgccctcttagagagcgtcactaagttccactgc                                                                                                                                                                                        >  1:341324‑M1/1‑185 (MQ=255)`  
                                                                                                                     `gCCTAACCTTGTCCTCGTTCGTATTCTCAAGTGCCGCTTTACTGGTGATACTGGTATCGCTGGCTACATGGAATACAACAAGGAAACCGGATGGCTTGAACCATCAAGTTACTCAGGGGAAGAAGAGTCACACTCAGAGTCAACAGACTGGTCCAACGACACTGACTTCTGACAGGATTCTTGAcagttgtttcatatgaagagattgttaagtcacgataatcaataggagaaatcaatatgatcgtttctgacatcgaagctaacgccctcttagagagctgtctcttatacacatctc                                                                                                                                                                                       >  1:308044‑M1/1‑184 (MQ=255)`  
                                                                                                                     `gCCTAACCTTGTCCTCGTTCGTATTCTCAAGTGCCGCTTTACTGGTGATACTGGTATCGCTGGCTACATGGAATACAACAAGGAAACCGGATGGCTTGAACCATCAAGTTACTCAGGGGAAGAAGAGTCACACTCAGAGTCAACAGACTGGTCCAACGACACTGACTT‑‑‑‑‑‑‑‑‑‑‑‑TTGAcagttgtttcatatgaagagattgttaagtcacgataatcaataggagaaatcaatatgatcgtttctgacatcgaagctaacgccctcttagagagcgtcactaagttccactgcggggttatctacg                                                                                                                                                                           >  1:77672‑M1/1‑172 (MQ=255)`  
                                                                                                                         `aaCCTTGTCCTCGTTCGTATTCTCAAGTGCCGCTTTACTGGTGATACTGGTATCGCTGGCTACATGGAATACAACAAGGAAACCGGATGGCTTGAACCATCAAGTTACTCAGGGGAAGAAGAGTCACACTCAGAGTCAACAGACTGGTCCAACGACACTGACTTCTGACAGGATTCTTGAcagttgtttcatatgaagagattgttaagtcacgataataaataggagaaatcaatatgatcgtttctgacatcgaagctaacgccctcttagagatgtctcatatacacatccgagcccc                                                                                                                                                                                   >  1:53130‑M1/1‑180 (MQ=255)`  
                                                                                                                          `aCCTTGTCCTTGTTCGTATTCTCAAGTGCCGCTTTACTGGTGATACTGGTATCGCTGGCTACATGGAATACAACAAGGAAACCGGATGGCTTGAACCATCAAGTTACTCAGGGGAAGAAGAGTCACACTCAGAGTCAACAGACTGGTCCAACGACACTGACTTCTGACAGGATTCTTGAcagttgtttcatatgaagagattgttaagccacgataatcaataggagaaatcaatatgatcgtttctgacatcgaagctaacgccctcttagagagcgtcactaagttccacctgtctctt                                                                                                                                                                                  >  1:35610‑M1/1‑179 (MQ=255)`  
                                                                                                                          `aCCTTGTCCTCGTTCGTATTCTCAAGTGCCGCTTTACTGGTGATACTGGTATCGCTGGCTACATGGAATACAACAAGGAAACCGGATGGCTTGAACCATCAAGTTACTCAGGGGAAGAAGAGTCACACTCAGAGTCAACAGACTGGTCCAACGACACTGACTTCTGACAGGATTCTTGATGACTTTCCAGACGACTACGAGAAGTTTCGCTGGAGAGTCCCATTCTAATACGACTCACTAAAGAAGACACACCATGTTCAAACTGATTAAGAAGGTAGGCCAACTGCTTGTTAGTATGTAc                                                                                                                                                                                  >  1:227684/1‑301 (MQ=255)`  
                                                                                                                                   `ggtgggctcggagatgtgtataagagacaggtgatactggtatcgctggctacatggaatACAACAAGGAAACCGGATGGCTTGAACCATCAAGTTACTCAGGGGAAGAAGAGTCACACTCAGAGTCAACAGACTGTTCCAACGACACTGACTTCTGACAGGATTCTTGAcagttgtttcatatgaagagattgttaagtcacgataatcaataggagaaatcaatatgatcgtttctgacatcgaagctaacgccatcttagagagcgtcactaagttccactgcggggttatctacgac                                                                                                                                                                         <  1:192450‑M1/272‑132 (MQ=255)`  
                                                                                                                                   `ccgttcgtaTTCTCAAGTTCCGCTTTACTGGTGATACTGGTTGCGCTGGCTACATGGAATACAACAAGGAAACCGGATTGCTTGAACCATCAAGTTACTCAGTGGAAGAAGAGTCACACTCAGATTCAACAGACTGGTCCAACGACACTGACTTCTGACATGATTCTTGAcagttgtgtcatatgaagagattgttaagtcacgataatcaataggagaaatcaatatgatcgtttctgacatcgaagctaacgccctctgagagagcgtcactaagttccactgctgggttatctacgac                                                                                                                                                                         <  1:270613‑M1/300‑132 (MQ=255)`  
                                                                                                                                    `cgttcgtATTCTCAAGTGCCGCTTTACTGGTGATACTGGTATCGCTGGCTACATGGAATACAACAAGGAAACCGGATGGCTTGAACCATCAAGTTACTCAGGGGAAGAAGAGTCACACTCAGAGTCAACAGACTGGTCCAACGACACTGACTTCTGACAGGATTCTTGAcagttgtttcatatgaagagattgttaagtcacgataatcaataggagaaatcaatatgatcgtttctgacatcgaagctaacgccctcttagagagcgtcactaactgtctcttatacacatctccgagcc                                                                                                                                                                        >  1:113277‑M1/1‑169 (MQ=255)`  
                                                                                                                                    `cgttcgtATTCTCAAGTGCCGCTTTACTGGTGATACTGGTATCGCTGGCTACATGGAATACAACAAGGAAACCGGATGGCTTGAACCATCAAGTTACTCAGGGGAAGAAGAGTCACACTCAGAGTCAACAGACTGGTCCAACGACACTGACTTCTGACAGGATTCTTGAcagttgtttcatatgaagagattgttaagtcacgataatcaaacaggagaaatcaatatgatcgtttctgacatcgaagctaacgccctcttagagagcgtcactaagttccactgcggggttatctacgac                                                                                                                                                                        <  1:203973‑M1/301‑133 (MQ=255)`  
                                                                                                                                      `ttcgtATTCTCAAGTGCCGCTTTACTGGTGATACTGGTATCGCTGACTACATGGAATACAACAAGGAAACCGGATGGCTTGAACCATCAAGTTACTCAGGGGAAGAAGAGTCACACTCAGAGTCAACAGACTGGTCCAACGACACTGACTTCTGACAGGATTCTTGAcagttgtttcatatgaagagattgttaagtcacgataatcaataggagaaatcaatatgatcgtttctgacatcgaagctaacgccctcttagagagcgtcactaagttcaactgcggggttatctacgactac                                                                                                                                                                      >  1:94983‑M1/1‑167 (MQ=255)`  
                                                                                                                                            `ttCTCAAGTGCCGCTTTACTGGTGATACTGGTATCGCTGGCTACATGGAATACAACAAGGAAACCGGATGGCTTGAACCATCAAGTTACTCAGGGGAAGAAGAGTCACACTCAGAGTCAACATACTGGTCCAACGCCACTGACTTCTGACAGGATTCTTGATGACTTTCCAGACGACTACGAGAAGTTTCGCTGGGAAGTCCCATTCTAATACGACTCACTAAAGGAGACCCACCATGTTCAACCTGATTAAGAAGTAAGCCCAACTGCTGGTTCGTATGTACAACGTGGAAGCCAAGCgc                                                                                                                                                                >  1:115760/1‑300 (MQ=255)`  
                                                                                                                                             `tctcAAGTGCCGCTTTACTGGTGAGACTGGTATCGCTGGCTACATGGAATACAACAAGGAAACCGGATGGCTTGAACCATCAAGTTACTCAGGGGAAGAAGAGTCACACTCAGAGTCAACAGACTGGTCCAACGACACTGACTTCTGACAGGATTCTTGATGACTTTCCAGACGACTACGAGAAGTTTCGCTGGAGAGTCCCATTCTAATACGACTCACTAAAGGAGACACACCATGTTCAAACTGATTAAGAAGTTAGGCCAACTGCTGGTTCGTATGTACAACGTGGAAGCCAAGCGAc                                                                                                                                                               <  1:331153/301‑1 (MQ=255)`  
                                                                                                                                              `taagagacaggctttactggtGATACTGGTATCGCTGGCTACATGGAATACAACAAGGAAACCGGATGGCTTGAACCATCAAGTTACTCAGGGGAAGAAGAGTCACACTCAGAGTCAACAGACTGGTCCAACGACACTGACTTCTGACAGGATTCTTGAcagttgtttcatatgaagagattgttaagtcacgataatcaataggagaaatcaatatgatcgtttctgacatcgaagctaacgccctcttatagagcgtcactaagttccactgcggggttatctacgactactccaccgc                                                                                                                                                              <  1:280027‑M1/291‑143 (MQ=255)`  
                                                                                                                                              `ctcAAGTGCCGCTTTACTGGTGATACTGGTATCGCTGGCTACATGGAATACAACAAGGAAACCGGATGGCTTGAACCATCACGTTACTCAGGGGAAGAAGAGTCACACTCAGAGTCAACAGACTGGTCCAACGACACTGACTTCTGACAGGATTCTTGAcagttgtttcatatgaagagattgttaagtcacgataatcaataggagaaatcaatatgatcgtttctgacatcgaagctaacgccctcttagagagcgtcactacgttccactgcggggttatctacgactacctgtctct                                                                                                                                                              >  1:68534‑M1/1‑159 (MQ=255)`  
                                                                                                                                                     `gCCGCTTTACTGGTGATACTGGTATCGCTGGCTACATGGAATACAACAAGGAAACCGGATGGCTTGAACCATCAAGTTACTCAGGGGAAGAAGAGTCACACTCAGAGTCAACAGACTGGTCCAACGACACTGACTTCTGACAGGTTTCTTGAcagttgtttcatatgaagagattgttaagtcacgataatcaataggagaaatcaatatgatcgtttctgacatcgaagctaacgccctcttagagagcgtcactaagttccactgcggggttatctacgactactccaccgctgagtac                                                                                                                                                       <  1:238160‑M1/301‑150 (MQ=255)`  
                                                                                                                                                      `ccGCTTTACTGGTGATACTGGTATCGCTGGCTACATGGAATACAACAAGGAAACCGGATGGCTTGAACCATCAAGTTACTCAGGGGAAGAAGAGTCACACTCAGAGTCAACAGACTGGTCCAACGACACTGACTTCTGACAGGATTCTTGATGACTTTCCAGACGACTACGAGAAGTTTCGCTGGAGAGTCCCATTCTAATACGACTCACTAAAGGAGACACACCATGTTCAAACTGATTAAGAAGTTAGGCCAACTGCTGGTTCGTATGTACAACGTGGAAGCCAAGCGACTGAACGaaa                                                                                                                                                      >  1:248344/1‑299 (MQ=255)`  
                                                                                                                                                        `cagttACTGGTGATACTGGTATCGCTGGCTACATGGAATACAACAAGGAAACCGGATGGCTTGAACCATCAAGTTACTCAGGGGAAGAAGAGTCACACTCAGAGTCAACAGACTGGTCCAACGACACTTACTTCTGACATGATTCTTGATGACTTTCCAGACGACTACGAGAAGTTTCGCTGTAGAGTCCCATTCTAATACGACTCACTAAAGGAGACACACCATGTTCAAACTGATTAAGAAGTTAGGCCAACTGCTGGTTCGTATGTACAACGTGGAAGCCAAGCGACTGAACGATGAg                                                                                                                                                    <  1:143083/298‑1 (MQ=255)`  
                                                                                                                                                          `tttACTGGTGATACTGGTATCGCTGGCTACATTGAATACAACAAGGAAACCGGATGGCTTGAACCATCAAGTTACTCAGGGGAAGAAGAGTCACACTCAGAGTCAACAGACTGGTCCAACGACACTGACTTCTGACAGGATTCTTGAcagttgtttcatatgaagagcttgttaagtcacgataatcaataggagaaatcaatatgatcgtttctgacatcgaagccaacgccctcttagagagcgtaactaagttccactgcggggttatctacgactactccaccgctgagtacgtaag                                                                                                                                                  <  1:160174‑M1/301‑155 (MQ=255)`  
                                                                                                                                                                `gtgggctcggagatgtgtataagagacagatacaacaaggaaaccggatggcgttaaccATCAAGTTACTCAGGGGAAGAAGAGTCACACTCATAGTCAACAGACTGGTCCAACGACACTGACTTGTGACAGGATTCTTGAcagttgtttcatatgaagagattgttaagtcacgataatcaataggagaaatcaatatgatcgtttctgacatcgaagctaacgccctcttagagagcgtcactaagttccactgcggggttatctacgactactccaccgctgagtacgtaagctaccg                                                                                                                                            <  1:258279‑M1/272‑161 (MQ=255)`  
                                                                                                                                                                  `tGATACTGGTATCGCTGGCTACATGGAATACAACAAGGAAACCGGATGGCTTGAACCATCAAGTTACTCAGGGGAAGAAGAGTCACACTCAGAGTCAACAGACTGGTCCAACGACACTGACTTCTGACAGGATTCTTGATGACTTTCCAGACGACTACGAGAAGTTTCGCTGGAGAGTCCCATCCTAATACGACTCACTAAAGGAGACACACCATGTTCAAACTGATTAAGAAGTTAGGCCAACTGCTGGTTCGTATGTACAACGTGGAAGCCAAGCGACTGAACGATGAGGCTCGTAAag                                                                                                                                          <  1:151315/301‑1 (MQ=255)`  
                                                                                                                                                                     `tACTGGTATCGCTGGCTACATGGAATACAACAAGGAAACCGGATGGCTTGAACCATCAAGTTACTCAGGGGAAGAAGAGTCACACTCAGAGTCAACAGACTGGTCCAACGACACTGACTTCTGACAGGATTCTTGAcagttgtttcatatgaagagattgttaagtcacgataatcaataggagaaatcaatatgatcgtttctgacatcgaagctaacgccctcttagagagcgtcactaagttccactgcggggttatctacgactactccacagctgagtacgtaagctaccgtccga                                                                                                                                       >  1:261487‑M1/1‑136 (MQ=255)`  
                                                                                                                                                                     `tACTGGTATCGCTGGCTACATGGAATACAACAAGGAAACCGGATGGCTTGAACCATCAAGTTACTCAGGGGAAGAAGAGTCACACTCAGAGTCAACAGACTGGTCCAACGACACTGACTTCTGACAGGATTCTTGATGACTTTCCAGACGACTACGAGAAGTTTCGCTGGAGAGTCCCATTCTAATACGACTCACTAAAGGAGACACACCATGTTCAAACTGATTAAGAAGTTAGGCCAACTGCTGGTTCGTATGTACAACGTGGAAGCCAAGCGACTGAACGATGAGGCTCGTAAAGAgg                                                                                                                                       >  1:324418/1‑301 (MQ=255)`  
                                                                                                                                                                         `ggTATCGCTGGCTACATGGAATACAACAAGGAAACCGGATGGCTTGAACCATCAAGTTACTCAGGGGAAGAAGAGTCACACTCAGAGTCAACAGACTGGTCCAACGACACTGACTTCTGACAGGATTCTTGAcagttgtttcatatgaagagattgttaagtcacgataatcaataggagaaatcaatatgatcgtttctgacatcgaagctaacgccctcttagagagcgtcactaagttccactgcgtggttatctacgactactccaccgctgagtacgtaagctaccgtccgagtga                                                                                                                                   >  1:276206‑M1/1‑132 (MQ=255)`  
                                                                                                                                                                         `ggTATCGCTGGCTACATGGAATACAACAAGGAAACCGGATGGCTTGAACCATCAAGTTACTCAGGGGAAGAAGAGTCACACTCAGAGTCAACAGACTGGTCCAACGACACTGACTTCTGACAGGATTCTTGAcagttgtttcatatgaagagattgttaagtcacgataatcaataggagaaatcaatatgatcgtttctgacatcgaagctaacgccctcttagagagcgtcactaagttccactgcggggttatctacgactactccaccgctgagtacgtaagctaccgtccgagtga                                                                                                                                   >  1:267655‑M1/1‑132 (MQ=255)`  
                                                                                                                                                                                `gagacatcatggaatACAACAAGGAACCCGGATGGCTTGAACCATCAAGTTACTCAGGGGAAGAAGTGTCACACTCAGAGTCAACAGACTGGTCCAACGACACTGACTT‑‑‑‑‑‑‑‑‑‑‑‑TTGAcagttgtttcatatgaagagattgttaagtcacgataatcaataggagaaatcaatatgatcgtttctgacatcgaagctaacgccctcttagagagcgtcactaagttccactgcggggttatctacgactactccaccgctgagtacgtaagctaccgtccgagtgacttcggtgcgtatctggat                                                                                                                <  1:290496‑M1/294‑189 (MQ=255)`  
                                                                                                                                                                                  `ggCTACATGGAATACAACAAGGAAACCGGATGGCTTGAACCATCAAGTTACTCAGGGGAAGAAGAGTCACACTCAGAGTCAACAGACTGGTCCAACTACACTTACTTCTGACAGGATTCTTGATGACTTTCCAGACGACTACGAGAAGTTTCGCTGGAGAGTCCCATTCTAATACGACTCACTAAAGGAGACACACCATGTTCAAACTGATTAAGAAGTTAGGCCAACTGCTGGTTCGTATGTACAACGTGGAAGCCAAGCGACTGAACGATGAGGATCGTaactgtctcttatacacatc                                                                                                                          >  1:252750/1‑283 (MQ=255)`  
                                                                                                                                                                                       `gagatgtgtataaggtacagccggatgtcttgaaccatcaagTTACTCAGGGGAAGAAGAGTCACACTCAAAGTCAACACACTGGTCCAACGACACTGACTTCTGACAGGATTCTTGAcagttgtttcatacgaagagattgttaagtcacgataatcaataggagaaatcaatatgatcgtttctgacatcgaagctaacgccctcttagagagcgtcactaagttccactgcggggttatctacgactactccaccgctgagtacgtaagctaccgtccgagtgacttcggtgcgtatc                                                                                                                     <  1:118158‑M1/281‑184 (MQ=255)`  
                                                                                                                                                                                           `gAATACAACAAGGAAACCGGATGGCTTGAACCATCAAGTTACTCAGGGGAAGAAGAGTCACACTCAGAGTCAACAGACTGGTCCAACGACACTGACTTCTGACAGGATTCTTGAcagttgtttcatatgaagagattgttaagtcacgataatcaataggagaaatcaatatgatcgtttctgacatcgaagctaacgccctcttagagagcgtcactaagttccactgcggggttatctacgactactccaccgctgattacgtaagctaccgtccgagtgacttcggtgcgtatcctgt                                                                                                                 >  1:327467‑M1/1‑114 (MQ=255)`  
                                                                                                                                                                                           `gAATACAACAAGGAAACCGGATGGCTTGAACCATCAAGTTACTCAGGGGAAGAAGAGTCACACTCAGAGTCAACAGACTGGTCCAACGACACTGACTTCTGACAGGATTCTTGAcagttgtttcatatgaagagattgttaagtcacgataatcaataggagaaatcaatatgatcgtttctgacatcgaagctaacgccctcttagagagcgtcactaagttccactgcggggttatctacgactactccaccgctgagtacgtaagctaccgtccgagtgacttcggtgcgtatctgga                                                                                                                 <  1:153722‑M1/301‑188 (MQ=255)`  
                                                                                                                                                                                           `gAATACAACAAGGAAACCGGATGGCTTAAACCATCAAGTTACTCAGGGGAAGAAGAGTCACACTCAGAGTCAACAGACTGGTCCACCGACCCTGACTTCTGACAGGATTCTTGATGCCTTTCCAGACAACTACAAGAATTTTCGCTGGAGAGTCCCATTCTAATACGACTCCCTAAAGGAGACACCCCATGTTCAAACGGATTACGAAGTTAGGCAACCTCCTGTTTCTTAGTTACAACGTGGAAGCAAAGCGACTGACCGAGAAGGCTCGTAAAGAGGCCCCACATTccctgtctcttat                                                                                                                 >  1:39476/1‑289 (MQ=255)`  
                                                                                                                                                                                            `aaTACAACAAGGAAACCGGATGGCGTGAACCATCAAGTTACTCAGGGGAAGAAGAGTCAAACTCAGAGTCAACAGACTGGTCCAACGACACTGACTTCTGACAGGATTCTTGAcagttgtttcatatgaagagattgttaagtcacgataatcaataggagaaatcaatatgatcgtttctgacatcgaagctaacgccctcttagagagcgtcactaagttccactgcggggttatctacgactactccaccgctgagtacgtaagctaccgtccgagtgacttcggtgcgtatctggat                                                                                                                <  1:196986‑M1/301‑189 (MQ=255)`  
                                                                                                                                                                                              `aacaacaAGGAAACCGGATGGCTTGAACCATCAAGTTACTCAGGGGAAGAAGAGTCACACTCAGAGTCAACAGACTGGTCCAACGACACTGACTTCTGACAGGATTCTTGATGACTTTCCAGACGACTACGAGAAGTTTCGCTGGAGAGTCCCATTCTAATACGACTCACTAAAGGAGACACACCATGTTCAAACTGATTAAGAAGTTAGGCCAACTGCTGGTTCGTATGTACAACGTGGAAGCCAAGCGACTGAACGATGAGGCTCGTAAAGAGGCCACACAGTCAcgataatcaatagg                                                                                                              <  1:311220/300‑13 (MQ=255)`  
                                                                                                                                                                                                `caacaaGGAAACCGGATGGCTTGAACCATCAAGTTACTCAGGGGAAGAAGAGTCACACTCAGAGTCAACAGACTGGTCCAACGACACTGACTTCTGACAGGATTCTTGAcagttgtttcatatgaagagattgttaagtcacgataatcaataggagaaatcaatatgatcgtttctgacatcgaagctaacgccctcttggagagcgtcactaagttccactgcggggttatctacgactactccaccgctgagtacgtaagctaccgtccgagtgacttcggtgcgtatctggatctgt                                                                                                            >  1:169251‑M1/1‑109 (MQ=255)`  
                                                                                                                                                                                                           `ccGGATGGCTTGAACCATCAAGTTACTCAGGGGAAGAAGAGTCACACTCAGAGTCAACAGACTGGTCCAACGACACTGACTTCTGACAGGATTCTTGATGACTTTCCAGACGACTACGAGAAGTTTCGCTGGAGAGTCCCATTCTAATACGACTCACTAAAGGAGACACACCATGTTCAAACTGATTAAGAAGTTAGGCCAACTGCTGGTTCGTATGTACAACGTGGAAGCCAAGCGACTGAACGATGAGGCTCGTAAAGAGGCCACACAGTCAcgataatcaataggagaaatcaatatg                                                                                                 >  1:146652/1‑276 (MQ=255)`  
                                                                                                                                                                                                             `ggATGGCTTGAACCATCAAGTTACTCAGGGGAAGAAGAGTCACACTCAGAGTCAACAGACTGGTCCAACGACACTGACTTCTGACAGGATTCTTGAcagttgtttcatatgaagagattgttaagtcacgataatcaataggagaaatcaatatgatcgtttctgacatcgaagctaacgccctcttagagagcgtcactaagttccactgcggggttatctacgactactccaccgctgagtacgtaagctaccgtccgagtgacttcggtgcgtatctggatgcgctggaagccgaggt                                                                                               <  1:215333‑M1/301‑206 (MQ=255)`  
                                                                                                                                                                                                                 `ggCTTGAACCATCAAGTTACTCAGGGGAAGAAGAGTCACACTCAGAGTCAACAGACTGGTCCAACGACACTGACTTCTGACAGGATTCTTGAcagttgtttcatatgaagagattgttaagtcacgataatcaataggagaaatcaatatgatcgtttctgacatcgaagctaacgccctcttagagagcgtcactaagttccactgcggggttatctacgactactccaccgctgagtacgtaagctaccgtccgagtgacttcggtgcgtatctggatgcgctggaagccgaggttgca                                                                                           >  1:273391‑M1/1‑92 (MQ=255)`  
                                                                                                                                                                                                                        `aCCATCAAGTTACTCATGGGAAGAAGAGTCACACTCAGAGTCAACAGACTGGTCCAACGACACTGACTTCTGACAGGATTCTTGAcagttgtttcatatgaagagattgttaagtcacgataatcaataggagaaatcaatatgatcgtttctgacatcgaagctaacgccctcttagagagcgtcactaagttccactgcggggttatctacgactactccaccgctgagtacgtaagctaccgtccgagtgacttcgatgcgtatctggatgcgctggaagccgaggttgcacgaggcg                                                                                    >  1:190180‑M1/1‑85 (MQ=255)`  
                                                                                                                                                                                                                                     `tCAGGGGAAGAAGAGTCACACTCAGAGTCAACAGACTGGTCCAACGACACTGACTTCTGACAGGATTCTTGAcagttgtttcatatgaagagattgttaagtcacgataatcaatagtagaaatcaatatgatcgtttctgacatcgaagccaacgccctcttagagagcgtcactaagttccactgcggggttatctacgactactccaccgctgagtacgtaagctaccgtccgagtgacttcggtgcgtatctggatgcgctggaagccgaggttgcacgaggcggtcttattgtgtt                                                                       >  1:224315‑M1/1‑72 (MQ=255)`  
                                                                                                                                                                                                                                         `ggaaataataatcacactcagagTCAACAGACTGGACCAACGACACTAACTTCTGACAGGAGTCTTGAcagttgtttcatatgaatagattgttacgtcacgataatcaataggagaaatcaatatgatcgtttctgacatcgaagccaacgcactcttatagagcgtcactaagttacactgcggggttatctacgactactccaccgctgagtacgtaagctaccgtccgagtgacttcggtgcgtatctggatgcgctggaagccgaggttgcacgaggcggtcttattgtgttccac                                                                   <  1:300072‑M1/290‑234 (MQ=255)`  
                                                                                                                                                                                                                                           `gaagaaGAGTCACATTCAGAGTCAACAGACTGGTCCAACGACACTGACGTCTGACAGGATTCTTGAcagttgtttcatatgaagagattgttaagtcacgataatcaataggagaaatcaatatgatcgtttctgacatcgaagctaacgccctcttagagagcgtcactaagttccactgcggggttatctacgactactccaccgctgagtacgtaagctaccgtccgagtgacttcggtgcgtatctggatgcgctggaagccgaggttgcacgaggcggtcttattgtgttccacaa                                                                 <  1:243489‑M1/301‑236 (MQ=255)`  
                                                                                                                                                                                                                                           `gaagaaGAGTCACACTCAGAGTCAACAGACTGGTCCAACGCCACTGACTTCTGACAGGATTCTTGAcagttgtttcatatgaagagattgttaagtcacgataatcaataggagaaatcaatatgatcgtttctgacatcgaagctaacgccctcttagagagcgtcactaagttccactgcggggttatctacgactactccaccgctgagtacgtaagctaccgtccgagtgacttcggtgcgtatctggatgcgctggaagccgaggttgcacgaggcggtcttattgtgttccacaa                                                                 <  1:158242‑M1/301‑236 (MQ=255)`  
                                                                                                                                                                                                                                              `gaagaGTCACACTCAGAGTCAACAGACTGGTCCAACGACACTGACTTCTGACAGGATTCTTGAcagttgtttcatatgaagagattgttaagtcacgataatcaataggagaaatcaatatgatcgtttctgacatcgaagctaacgccctcttagagagcgtcactaagttcaactgcggggttatctacgactactccaccgctgagtacgtaagctaccgtccgagtgacttcggtgcgtatctggatgcgctggaagccgaggttgcacgaggcggtcttattgtgttccacaacgg                                                              >  1:338706‑M1/1‑63 (MQ=255)`  
                                                                                                                                                                                                                                                   `gTCACACTCAGAGTCAACAGACTGGTCCAACGACACTGACTTCTGACAGGATTCTTGAcagttgtttcatatgaagagattgttaagtcacgataatcaataggagaaatcaatatgatcgtttctgacatcgaagctaacgccctcttagagagcgtcactaagttccactgcggggttatctacgactactccaccgctgagtacgtaagctaccgtcctagtgccttcggtgcgtatctggatgcgctggaagccgaggttgcacgaggcggtcttattgctgtctcttatacacatc                                                         >  1:254777‑M1/1‑58 (MQ=255)`  
                                                                                                                                                                                                                                                          `tCAGAGTCAACAGACTGGTCCAACGACACTGACTTCTGACAGGATTCTTGAcagttgtttcatatgaagagattgttaagtcacgataatcaataggagaaatcaatatgatcgtttctgacatcgaagctaacgccctcttagagagcgtcactaagttccactgcggggttatctacgactactccaccgctgagtacgtaagctaccgtccgagtgacttcggtgcgtatctggatgcgctggaagccgaggttgcacgaggcggtcttattgtgttccacaacggtcacaagtatga                                                  <  1:218352‑M1/301‑251 (MQ=255)`  
                                                                                                                                                                                                                                                             `gtataagagacagggtccaacgacactgACTTCTGACAGGATTCTTGAcagttgtttcatatgaagagattgttaagtcacgataatcaataggagaaatcaatatgatcgtttctgacatcgaagctaacgccctcttagagagcgtcactaagttccactgcggggttatctacgactactccaccgctgagtacgtaagctaccgtccgagtgacttctgtgcgtatctggatgcgctggaagccgaggttgcacgaggcggtcttattgtgttccacaacggtcacaagtatgacgt                                               <  1:182889‑M1/288‑254 (MQ=255)`  
                                                                                                                                                                                                                                                             `aagtCAACAGACTGGTCCAACGACACTGACTTCTGACAGGATTCTTGAcagttgtttcatatgaagagattgttaagtcacgataatcaataggagaaatcaatatgatcgtttctgacatcgaagctaacgccctcttagagagcgtcactaagttccactgcggggttatctacgactactccacagctgagtacgtaagctaccgtccgagtgacttcggtgcgtatctggatgcgctggaagccgaggttgcacgaggcggtcttattgtgttccacaacggtcacaagtatgacgt                                               <  1:171106‑M1/300‑254 (MQ=255)`  
                                                                                                                                                                                                                                                               `gTCAACAGACTGGTCCAACGACACTGACTTCTGACAGGTTTCTTGAcagttgtttcatatgaagagattgttaagtcacgataatcaataggagaaatcaatatgatcgtttctgacatcgaagctaacgccctcttagagagcgtcactaagttccactgcggggttatctacgactactccaccgctgagtacgtaagctaccgtccgagtgacttcggtgcgtatctggatgcgctggaagccgaggttgcacgaggcggtcttattgtgttccacaacggtcacaagtatgactgtc                                             >  1:103310‑M1/1‑46 (MQ=255)`  
                                                                                                                                                                                                                                                                       `aCTGGTCCAACGACACTGACTTCTGACAGGTTTCTTGATGACTTTCCAGACGACTACGAGAAGCTTCGCTGGAGAGTCCCATTCTAATACGACTCACTAAAGGAGACACACCATGTTCAAACTGATTAAGAAGTTAGGCCAACTTCTGGTTCGTATGTACAACGTGGAAGCCAAGCGACTGAACGATGAGGCTCGTAAAGAGGCCACACAGTCACGCGCTCTGGCGATTCGCTCCAACGAACTGGCTGACAGTGCATCCACTAAAGTTACCGAGGCTGCCCGTGTGGCAAACCAAGCTctg                                     <  1:282153/301‑3 (MQ=255)`  
                                                                                                                                                                                                                                                                           `gTCCAACGACACTGACTTCTGACAGGATTCTTGAcagttgtttcatatgaagagattgttaagtcacgataatcaataggagaaatcaatatgatcgtttctgacatcgaagctaacgccctcttagagagcgtcactaagttccactgcggggttatctacgactactccaccgctgagtacgtaagctaccgtccgagtgacttcggtgcgtatctggatgcgctggaagccgaggttgcacgaggcggtcttattgtgttccacaccggtcaaaagtatgacgttcctgcatcgacca                                 >  1:85899‑M1/1‑34 (MQ=255)`  
                                                                                                                                                                                                                                                                                                    `gATTCTTGAcagttgtttcatatgaagagattgttaagtcacgataatcaataggagaaatcaatatgatcgtttctgacatcgaagctaacgccctcttagagagcgtcactaagttccactgcggggttatctacgactactccaccgctgagtacgtaagctaccgtccgagtgacttcggtgcgtatctggatgcgctggaagccgaggttgcacgaggcggtcttattgtgttccacaacggtcacaagtatgacgttcctgcattgaccaaactggcaaagttgcaattgaaccg        <  1:97619‑M1/301‑293 (MQ=255)`  
                                                                                                                                                                                                                                                                                                      `ttCTTGAcagttgtttcatatgaagagattgttaagtcacgataatcaataggagaaatcaatatgatcgtttctgacatcgaagctaacgccctcttagagagcgtcactaagttccactgcggggttatctacgactactccaccgctgagtacgtaagctaccgtccgagtgacttcggtgcgtatctggatgcgctggaagccgaggttgcacgaggcggtcttattgtgttccacaacggtcacaagtatgacgttcctgcattgaccaaactggcaaagttgcaattgaaccgag      >  1:285510‑M1/1‑7 (MQ=255)`  
                                                                                                                                                                                                                                                                                                        `cTTGATGACTTTCCAGACGACTACGAGAAGTTTCGCTGTAGAGTCCCATTCTAATACGACTCACTAAAGGAGACACACCATGTTCAAACTGATTAAGAAGTTAGGCCAACTGCTGGTTCGTATGTACAACGTGGAAGCCAAGCGACTGAACGATGAGGCTCGTAAAGAGGCCACACAGTCACGCGCTCTGGCGATTCGCTCCAACTAACTGGCTGACAGTGCATCCACTAAAGTTACCGAGGCTGCCCGTGTGGCAAACCAAGCTCAACAGCTTTCCAAATTCTTTGAGTAATCAAACAgg    >  1:211474/1‑301 (MQ=255)`  

                                                                                                                                                                                                                                                                                                            `|`                                                                                                                                                                                                                                                                                                            
`AGGTAAAGCACATGAGGAAGGTCGCCCCGTTTCTATTACTGACCTACGTGGTTCTGGCGCACTACGCCAACTATCTGATACTATTATTGCCCTTGAGCGTAATCAGCAAGGCGATATGCCTAACCTTGTCCTCGTTCGTATTCTCAAGTGCCGCTTTACTGGTGATACTGGTATCGCTGGCTACATGGAATACAACAAGGAAACCGGATGGCTTGAACCATCAAGTTACTCAGGGGAAGAAGAGTCACACTCAGAGTCAACAGACTGGTCCAACGACACTGACTTCTGACAGGATTCTTGATGACTTTCCAGACGACTACGAGAAGTTTCGCTGGAGAGTCCCATTCTAATACGACTCACTAAAGGAGACACACCATGTTCAAACTGATTAAGAAGTTAGGCCAACTGCTGGTTCGTATGTACAACGTGGAAGCCAAGCGACTGAACGATGAGGCTCGTAAAGAGGCCACACAGTCACGCGCTCTGGCGATTCGCTCCAACGAACTGGCTGACAGTGCATCCACTAAAGTTACCGAGGCTGCCCGTGTGGCAAACCAAGCTCAACAGCTTTCCAAATTCTTTGAGTAATCAAACAGGAG  >  NC_001604.1/12977‑13575`

  

Alignment Legend

Aligned base mismatch/match (shaded by quality score): `ATCG/ATCG` `< 3 ≤` `ATCG/ATCG` `< 11 ≤` `ATCG/ATCG` `< 25 ≤` `ATCG/ATCG` `< 36 ≤` `ATCG/ATCG` `< 38 ≤` `ATCG/ATCG`

Unaligned base: `atcg`    Masked matching base: `atcg`    Alignment gap: `‑`    Deleted base: `‑`

Reads not counted as support for junction

read\_name Not counted due to insufficient overlap past the breakpoint.

read\_name Not counted due to not crossing MOB target site duplication.
